# Heterogeneity in corporate payouts

**DOI:** 10.1016/j.heliyon.2023.e23270

**Published:** 2023-12-06

**Authors:** Adhiraj Sodhi, Aleksandar Stojanovic

**Affiliations:** The Business School, University of Greenwich, London, UK

**Keywords:** Dividends, Repurchases, Determinants

## Abstract

In this paper we empirically investigate dividends and repurchases of the UK, and the overarching pattern reveals that their determinants are more driven by payout size than they are by payout type. The overall corporate payout policy is influenced by the operating performance and tax framework. Aggregately, the determinants of dividends and repurchases are heterogenous, and for each payout's individual testing the determinants have shown varying influences when controlled for payout size; small, medium and large. Comparatively, the determinants of dividends and repurchases of small size show homogeneity, while those of medium and large sizes exhibit heterogeneity. From a variable-specific perspective, aggregately dividends are positively influenced by asset holdings and ROA, and negatively influenced by independent directions and EPS. While aggregately repurchases are positively influenced by debt exposure and negatively influenced by M/B Ratio.

## 1 Introduction

Dividends and repurchases are two sides of the same corporate payout coin, and when firms decide that cash must be distributed amongst shareholders, they can choose either or both. The US is the world leader in repurchases, but the UK too has seen a rise in relative popularity that makes it second in the world [1,2]. Despite the two countries fully legalising open market repurchases around the same time, the UK in 1981 [3] and US in 1982 [4], by 2000 the repurchase-dividend ratio in the US increased from 13 % to over 110 % [5], while from 1989 to 2005 the ratio in the UK increased from 14 % to over 50 % [6]. The proportion of firms opting for repurchases in the UK increased from 4 % in 1992 to 15 % in 2004 [7]. Following this, the upward trend is witnessed in absolute terms [8] with annual value-level peaks of £33bn seen in 2006, again in 2018, and surpassing £55bn in 2022, which is still less than the £83bn worth of dividends expected to be distributed in 2023. In the UK the two payouts are viewed as independent from each other instead of being in a predatory relationship to replace each other [9,10,11]. These are voluminous payouts undertaken in a country that houses Europe's largest stock market [12], is the continent's second largest economy [13], and is one of the top OECD countries with respect to the level of corporate payouts [14], thus making it prime for an empirical examination.

It is arguable that if repurchases values are rising, then these are funded by diverting cash that would otherwise finance, and potentially increase, dividend distributions. However, the inherent cash reserves of British firms are also increasing, which enables optimal financing for both payouts. From 1994 to 2013, their reserves have jumped by 50 % [15], and between March 2019 to May 2021 the absolute value has increased from £20bn to £109bn [16].

In terms of the dissimilarity between the payouts’ fundamentals, dividends are negatively related to information asymmetry [17] while repurchases are positively related [18] and hence more able to offset information bias [19]. Furthermore, repurchases have certain advantages over dividends, such as being commitment-free [7], the ability to optimise the capital structure [20], and offsetting dilution due to employee compensation schemes triggering stock options [9]. The preference for the payouts differs due to factors such as the type of stakeholder and company-specific conditions. Blockholders in general are against undertaking any kind of payout to avoid cash shortages [7], and CEOs share this viewpoint but only for dividends [21]. However, dividends also cater to a specific class of investors that are looking for steady income, such as pension funds [22]. Further, post-IPO firms are more likely to commence their payout policy with repurchases [23]. This is attributable to repurchases being a safe investment outlet, and the opportunity for early investors to harvest capital gains. Dividend-led initiation is attributable to the market putting a dividend-expectation-premium on the share price, which is less likely for a young PLC.

Despite being dissimilar in their nature of delivering cash to shareholders and being independent in the UK, since they deliver the same function using cash from the same entity, they will always have some philosophical similarities. Both are largely pro-cyclical in nature [24,25,26,27,28], with some evidence that banking sector dividends [29] and tender offer repurchases [30] are counter-cyclical, which complements the ability of dividends to signal a positive earnings future [31] and repurchase's ability of adjusting the EPS [32]. They are also similar in terms of having the signalling ability of adjusting the stock valuation [33,34], and the ability of distributing surplus cash to limit agency conflicts [35,36].

Hence, given the combination of their foundational similarities which cannot be fully disentangled, and the variance in their practical applications, we investigate both payouts for the UK over a 30year period (1985–2014). Potential heterogeneity in their determinants is the empirical cornerstone, which is assessed using a set of 10 hypotheses. We first check if there is heterogeneity within each payout when we control for their size, Small (25th percentile), Medium (50th percentile) and Large (75th percentile), and then compare the payouts against each other as well. Our uniqueness lays in the reliance of investigating heterogeneity using a combination of Tobit and Quantile Regression methodologies over a 30year period using an array of 10 independent variables, which in combination proxy firm-level traits, financial performance and the tax framework. Our findings contribute to extant literature by establishing that heterogeneity in the determinants of corporate payouts is primarily a function of payout size, and secondarily of payout type. Aggregately, the determinants of dividends and repurchases are heterogenous. A transitionary change in the determinants’ influence within the payout is seen as size increases. When the two payouts are compared, the determinants of small size dividends and repurchases are homogenous, making them substitutable, and heterogeneity becomes visible as the payout size increases to medium and large, making them independent and non-substitutable.

## 2 Data

Our testing compares dividends and repurchases, and we begin by compiling firms that have undertaken both payouts. We procured data by purchasing it from Alacra Inc, a data vendor affiliated with Thomson Reuters who gathered the information for us from the SDC Platinum database. This included a list of open market repurchase announcing firms in the UK from 1985 to 2014. In total there were 419 initial announcements, and we only use these due to them being more information enriched compared to the regulatory mandated transacitonary announcements or shareholder reapproval announcements [37]. For building the independent variables, firm-level data was manually extracted from annual filings, and taxation data was acquired from HMRC and general government archives. Due to gaps in historical data, we were unable to locate the accounts for 59 announcements, the sample has reduced to 360 announcements and this highlights key traits about the firms’ payout behaviour. From the summary of the payouts in Table 1, only 13 % firms reported a net loss and 11 % firms avoided dividend distributions in the most recent annual filings. However, 84 % of the repurchases exceeded the most recent annual dividends distribution. Thus, it is possible that dividends are only partially curtailed to fund an upcoming repurchase. The average number of outstanding shares intended for repurchase is 9.90 %, which is similar to the median, thus indicating an even distribution. This number is also similar to that seen in the past [38]. In Table 2 we summarise the independent variables used in the empirical testing.

**Table 1:** Univariate characteristics.

Panel I: Key Sample Characteristics				
Firms with Negative Net Profit	No (%):		45 (13)	
Non-Dividend Payers Prior to Repurchase Announcements	No (%):		39 (11)	
Repurchases Greater than Dividends	No (%):		301 (84)	
**Panel II: Sample Payout characteristics**				
		**Full Sample**	**25**th **Percentile**	**75**th **Percentile**
Total Payout (£mn)	Mean	661.33	2.49	2495.69
	Median	33.68	2.03	914.71
Dividend Value (£mn)	Mean	197.90	0.19	757.52
	Median	7.00	0.09	241.95
Repurchase Value (£mn)	Mean	463.43	1.50	1753.29
	Median	26.08	1.28	618.31
% Shares being Repurchased	Mean	9.90	3.30	14.99
	Median	10.00	3.56	14.99

**Table 2:** Definition of the independent variables.

Variable	Description
Debt Ratio	Ratio of the total book value of debt to the book value of shareholder equity
Firm Size	Natural logarithm of total £mn book value of assets
Board	Fraction of independent directors in the board
M/B Ratio	Ratio of the company's market value to the book value
ROA	Net profit relative to total asset value
EPS	£ net profit per outstanding ordinary share
Rep Tax	Higher capital gains tax rate
Div Tax	Higher dividend tax rate
Tax Interaction	Product of the Rep Tax and Div Tax *(Interaction Variable)*
Tax Friendly	Binary, which takes the value ‘1’ if repurchases are tax friendlier than dividends

Lower leverage positions have driven dividends [[39], [40]] and repurchases [9,41] in the UK, hence we include *Debt Ratio*. The size of a company has often fuelled the distributions of dividends [40] and repurchase undertaking [42], hence we include *Firm Size*. Investment is not compromised for undertaking repurchases and dividend distribution are sticky in order to provide consistency in cash streams to shareholders [43] and since shareholders approve repurchases [44], and dividends curb agency conflicts [36], to test the influence of independent directors we include *Board*. British firms have shown to use dividends and repurchases to signal a positive corporate financial future [32,45], thus we include *M/B Ratio* as well. Complementary to this, we capture the effect of profitability by using *ROA* and *EPS*. The former has shown to drive the payouts [7], while the latter determines dividends [45] and is cited by British managers as a metric targeted for inflating using repurchases [32]. The tax framework in the UK has been ever changing, with progressive increases in tax levied on dividends, and reductions in tax levied on capital gains yielded from repurchases. Upon looking at government archives, we have tabulated these rates in Table 3, which reveal that over the tested period we start with dividends being twice more tax friendlier than repurchases and end with repurchases being tax friendlier than dividends. A tax code is efficient if it does not tax payouts when they originate in the companies (such as withholding tax) [46], and the UK follows this approach. Combining these two aspects, we employ four proxies to test the tax code's influence, which will smooth out any ambiguity due to fluctuations; *Rep Tax*, Div *Tax*, *Tax Interaction*, and *Tax Friendly*.

**Table 3:** Corporate payout tax rates.

Effective From	Dividend Tax	Effective From	Capital Gains Tax
1981	15 %	1981	30 %
1993	25 %	1988	10 %–40 %
1999 up to 2014	25 %–30.60 %	2008	18 %
		2012 up to 2014	18 %–28 %

## 3 Hypotheses and methodology

### 3.1 Determinants of corporate payouts

Our aim is to check the presence of heterogeneity in the determinants of corporate payouts, and we address this from three dimension, which are quantified using specific hypotheses. The first dimension is testing if the determinants of aggregate dividends and repurchases are heterogenous (H1), and we employ Tobit regression (Equations (1), (2))) by left censoring at 0 %, a common approach used by past corporate payout studies [9,20].

H10 = There is homogeneity between the determinants of aggregate dividends and repurchases

H11 = There is heterogeneity between the determinants of aggregate dividends and repurchases

(1) $${Dividends}_{i,y}=\beta_{1}{\text{Debt}\;\text{Ratio}}_{y-1}+\beta_{2}{\text{Firm}\;\text{Size}}_{i,y-1}+\beta_{3}{\text{Board}}_{i,y-1}+\beta_{4}{M/B\;\text{Ratio}}_{y-1}+\beta_{5}{\text{ROA}}_{y-1}+\beta_{6}{\text{EPS}}_{y-1}+\beta_{7}{\text{Rep}\;\text{Tax}}_{y}+\beta_{8}{\mathrm{Div}\;\text{Tax}}_{y}++\beta_{9}{\text{Tax}\;\text{Interaction}}_{y}+\beta_{10}{\text{Tax}\;\text{Friendly}}_{y}+\varepsilon_{i,y}$$

(2) $${Repurchases}_{i,y}=\beta_{1}{\text{Debt}\;\text{Ratio}}_{y-1}+\beta_{2}{\text{Firm}\;\text{Size}}_{i,y-1}+\beta_{3}{\text{Board}}_{i,y-1}+\beta_{4}{M/B\;\text{Ratio}}_{y-1}+\beta_{5}{\text{ROA}}_{y-1}+\beta_{6}{\text{EPS}}_{y-1}+\beta_{7}{\text{Rep}\;\text{Tax}}_{y}+\beta_{8}{\mathrm{Div}\;\text{Tax}}_{y}++\beta_{9}{\text{Tax}\;\text{Interaction}}_{y}+\beta_{10}{\text{Tax}\;\text{Friendly}}_{y}+\varepsilon_{i,y}$$

*Dividends* is the ratio of the ordinary dividends to the net profit,^1^ *Repurchases* is the ratio of the % outstanding shares intended for repurchase to the free cash flow (ratio of the operating profit to total asset value).^2^ *i* refers to the firm = 1, 2, 3 … 360, *y* refers to the year = 1985 to 2014 (inclusive), $\varepsilon_{i,t}$ is the error term, and the independent variables are defined in Table 2. Total number of observations is 3960 per equation.

The second dimension is testing if the determinants are heterogenous within dividends (H2, H3, and H4) and repurchases (H5, H6, and H7) when controlled for their size; *Small* = 25th percentile, *Medium* = 50th percentile and *Large* = 75th percentile. These hypotheses are tested using Quantile Regression (Equations (3) and (4)), which has been employed in variations by past corporate payout studies [47,48,49].

H20 = There is homogeneity between the determinants of Small and Medium sized dividends

H21 = There is heterogeneity between the determinants of Small and Medium sized dividends

H30 = There is homogeneity between the determinants of Small and Large sized dividends

H31 = There is heterogeneity between the determinants of Small and Large sized dividends

H40 = There is homogeneity between the determinants of Medium and Large sized dividends

H41 = There is heterogeneity between the determinants of Medium and Large sized dividends

(3) $${Dividends}_{s,i,y}=\beta_{1}{\text{Debt}\;\text{Ratio}}_{y-1}+\beta_{2}{\text{Firm}\;\text{Size}}_{i,y-1}+\beta_{3}{\text{Board}}_{i,y-1}+\beta_{4}{M/B\;\text{Ratio}}_{y-1}+\beta_{5}{\text{ROA}}_{y-1}+\beta_{6}{\text{EPS}}_{y-1}+\beta_{7}{\text{Rep}\;\text{Tax}}_{y}+\beta_{8}{\mathrm{Div}\;\text{Tax}}_{y}++\beta_{9}{\text{Tax}\;\text{Interaction}}_{y}+\beta_{10}{\text{Tax}\;\text{Friendly}}_{y}+\varepsilon_{i,y}+\alpha$$

*Dividends* is the ratio of the ordinary dividends to the net profit*,* s *=* is the size of the payout, *Small*, *Medium* or *Large*, *i* refers to the firm = 1, 2, 3 … 360, *y* refers to the year = 1985 to 2014 (inclusive), $\varepsilon_{i,t}$ is the error term, α is the alpha, and the independent variables are defined in Table 2. Total number of observations is 3960.

H50 = There is homogeneity between the determinants of Small and Medium sized repurchases

H51 = There is heterogeneity between the determinants of Small and Medium sized repurchases

H60 = There is homogeneity between the determinants of Small and Large sized repurchases

H61 = There is heterogeneity between the determinants of Small and Large sized repurchases

H70 = There is homogeneity between the determinants of Medium and Large sized repurchases

H71 = There is heterogeneity between the determinants of Medium and Large sized repurchases

(4) $${Repurchases}_{s,i,y}=\beta_{1}{\text{Debt}\;\text{Ratio}}_{y-1}+\beta_{2}{\text{Firm}\;\text{Size}}_{i,y-1}+\beta_{3}{\text{Board}}_{i,y-1}+\beta_{4}{M/B\;\text{Ratio}}_{y-1}+\beta_{5}{\text{ROA}}_{y-1}+\beta_{6}{\text{EPS}}_{y-1}+\beta_{7}{\text{Rep}\;\text{Tax}}_{y}+\beta_{8}{\mathrm{Div}\;\text{Tax}}_{y}+\beta_{9}{\text{Tax}\;\text{Interaction}}_{y}+\beta_{10}{\text{Tax}\;\text{Friendly}}_{y}+\varepsilon_{i,y}+\alpha$$

*Repurchases* is the ratio of % shares intended for repurchase to the free cash flow (ratio of the operating profit to total asset value). s *=* is the size of the payout, *Small*, *Medium* or *Large*, *i* refers to the firm = 1, 2, 3 … 360, *y* refers to the year = 1985 to 2014 (inclusive), $\varepsilon_{i,t}$ is the error term, α is the alpha, and the independent variables are defined in Table 2. Total number of observations is 3960.

The third dimension is to use the findings from the Quantile Regressions (Equations (3) and (4)), and checking if there is heterogeneity between the payouts of the same sizes (H8, H8 and H10)

H80 = There is homogeneity between the determinants of Small sized dividends and repurchases

H81 = There is heterogeneity between the determinants of Small sized dividends and repurchases

H90 = There is homogeneity between the determinants of Medium sized dividends and repurchases

H91 = There is heterogeneity between the determinants of Medium sized dividends and repurchases

H100 = There is homogeneity between the determinants of Large sized dividends and repurchases

H101 = There is heterogeneity between the determinants of Large sized dividends and repurchases

### 3.2 Robustness testing

For reliability we replicate the Tobit and Quantile Regressions for the total corporate payout (Equations (5), (6))).

(5) $${Total\;Payout}_{i,y}=\beta_{1}{\text{Debt}\;\text{Ratio}}_{y-1}+\beta_{2}{\text{Firm}\;\text{Size}}_{i,y-1}+\beta_{3}{\text{Board}}_{i,y-1}+\beta_{4}{M/B\;\text{Ratio}}_{y-1}+\beta_{5}{\text{ROA}}_{y-1}+\beta_{6}{\text{EPS}}_{y-1}+\beta_{7}{\text{Rep}\;\text{Tax}}_{y}+\beta_{8}{\mathrm{Div}\;\text{Tax}}_{y}++\beta_{9}{\text{Tax}\;\text{Interaction}}_{y}+\beta_{10}{\text{Tax}\;\text{Friendly}}_{y}+\varepsilon_{i,y}$$

(6) $${Total\;Payout}_{s,i,y}=\beta_{1}{\text{Debt}\;\text{Ratio}}_{y-1}+\beta_{2}{\text{Firm}\;\text{Size}}_{i,y-1}+\beta_{3}{\text{Board}}_{i,y-1}+\beta_{4}{M/B\;\text{Ratio}}_{y-1}+\beta_{5}{\text{ROA}}_{y-1}+\beta_{6}{\text{EPS}}_{y-1}+\beta_{7}{\text{Rep}\;\text{Tax}}_{y}+\beta_{8}{\mathrm{Div}\;\text{Tax}}_{y}+\beta_{9}{\text{Tax}\;\text{Interaction}}_{y}+\beta_{10}{\text{Tax}\;\text{Friendly}}_{y}+\varepsilon_{i,y}+\alpha$$

*Total Payout* is the natural logarithm of the £mn value of the sum of dividend and repurchase, s *=* is the size of the payout, *Small*, *Medium* or *Large*, *i* refers to the firm = 1, 2, 3 … 360, *y* refers to the year = 1985 to 2014 (inclusive), $\varepsilon_{i,t}$ is the error term, α is the alpha, and the independent variables are defined in Table 2. Total number of observations is 3960 per equation.

## 4 Results

### 4.1 Determinants of corporate payouts

In Table 4, Table 5 we tabulate the results from the testing of aggregate (Equation (1)) and size-specific dividends (Equation (3)), respectively, and in Table 6, Table 7 we tabulate the results from the testing of aggregate (Equation (2)) and size-specific repurchases (Equation (4)), respectively. Aggregately, we see that there is diversity in the findings, as in the case of dividends, *Firm Size* and *ROA* have positive influences, while *Board* and *EPS* have negative influences; the latter being relatively mild. Hence, when the firm has a larger asset base and is generating good returns against it, they want to reward shareholders for their investment via dividends. However, independent directors are against this cash depletion, perhaps to cater to the payout apprehension of CEOs and large blockholders [21,7]. The managerial seem to also distribute dividends during times of lower *EPS*, complying with the payout's ability to signal future confidence [19], which gains credibility by the positive impacts of asset holdings and the returns made against them. With repurchases, positive influence is generated by *Debt Ratio* and *Rep Tax*, the latter being mild, but *M/B Ratio* has a negative influence. Hence, as repurchases skew the debt/equity split, the managerial capitalise on the pre-existing shareholder debt appetite, while often compensating for higher tax bills. Furthermore, repurchases are also devised for signalling stock undervaluation. Thus, we see heterogeneity between the determinants of the dividends and repurchases, and we accept the alternative hypothesis H1_1_.

**Table 4:** Determinants of dividend financing: Aggregate.

	I	II	III	IV	V	VI	VII	VIII	IX	X	XI	XII
Debt Ratio	−0.021 *(-1.23)*	−0.015 *(-0.91)*	−0.022 *(-1.29)*	−0.016 *(-0.95)*	−0.020 *(-1.20)*	−0.015 *(-0.92)*	−0.020 *(-1.18)*	−0.015 *(-0.88)*	−0.022 *(-1.28)*	−0.016 *(-0.94)*	−0.020 *(-1.22)*	−0.015 *(-0.88)*
Firm Size	0.080*** *(3.59)*	0.093*** *(3.94)*	0.084*** *(3.69)*	0.095*** *(3.98)*	0.084*** *(3.71)*	0.095*** *(4.01)*	0.080*** *(3.59)*	0.093*** *(3.91)*	0.086*** *(3.78)*	0.096*** *(4.03)*	0.080*** *(3.52)*	0.092*** *(3.87)*
Board	−0.607*** *(-2.61)*	−0.048** *(-2.03)*	−0.569*** *(-2.41)*	−0.465* *(-1.95)*	−0.556** *(-2.35)*	−0.423* *(-1.75)*	−0.592** *(-2.54)*	−0.467** *(-1.97)*	−0.517** *(-2.16)*	−0.433* *(-1.78)*	−0.610*** *(-2.59)*	−0.490** *(-2.05)*
M/B Ratio	0.0008 *(0.11)*	−0.001 *(-0.12)*	0.001 *(0.15)*	−0.0008 *(-0.11)*	0.0004 *(0.05)*	−0.0009 *(-0.12)*	0.0007 *(0.09)*	−0.001 *(-0.14)*	0.001 *(0.20)*	−0.001 *(-0.13)*	0.0008 *(0.11)*	−0.001 *(-0.14)*
ROA		1.092** *(2.35)*		1.067** *(2.28)*		1.043** *(2.23)*		1.093** *(2.35)*		1.032** *(2.20)*		1.105** *(2.37)*
EPS		−0.176* *(-1.89)*		−0.174* *(-1.87)*		−0.155 *(-1.64)*		−0.168* *(-1.78)*		−0.153 *(-1.62)*		−0.179* *(-1.91)*
Rep Tax			1.022 *(1.03)*	0.562 *(0.54)*					9.537 *(0.85)*	8.779 *(0.78)*		
Div Tax					−1.403 *(-1.22)*	−1.468 *(-1.21)*			10.363 *(0.71)*	9.846 *(0.67)*		
Tax Interaction							−2.368 *(-0.85)*	−1.997 *(-0.71)*	−31.000 *(-0.83)*	−28.866 *(-0.77)*		
Tax Friendly											0.012 *(0.08)*	0.049 *(0.30)*
Constant	−0.328 *(-1.10)*	−0.596* *(-1.88)*	−0.781 *(-1.47)*	−0.840 *(-1.53)*	−0.048 *(-0.13)*	−0.284 *(-0.70)*	−0.109 *(-0.28)*	−0.406 *(-0.98)*	−3.742 *(-0.84)*	−3.744 *(-0.85)*	−0.324 *(-1.07)*	−0.584* *(-1.83)*
Pseudo R^2^	0.019	0.029	0.020	0.029	0.020	0.031	0.019	0.030	0.024	0.038	0.019	0.029

Superscripts state the level of statistical significance = 1 % (***), 5 % (**) and 10 % (*), t-statistics are stated in the parenthesis.

**Table 5:** Determinants of dividend financing: Size-specific.

Panel I: Small Sized												
	I	II	III	IV	V	VI	VII	VIII	IX	X	XI	XII
Debt Ratio	−0.019*** *(-3.23)*	−0.014*** *(-2.58)*	−0.019*** *(-3.52)*	−0.015*** *(-2.95)*	−0.014** *(-2.51)*	−0.020*** *(-3.51)*	−0.017*** *(-3.04)*	−0.014** *(-2.22)*	−0.014*** *(-2.77)*	−0.023*** *(-3.98)*	−0.019*** *(-3.39)*	−0.015*** *(-2.60)*
Firm Size	0.049*** *(6.48)*	0.048*** *(6.25)*	0.048*** *(6.65)*	0.047*** *(6.48)*	0.041*** *(5.42)*	0.042*** *(5.47)*	0.048*** *(6.34)*	0.047*** *(5.36)*	0.040*** *(5.96)*	0.041*** *(5.26)*	0.048*** *(6.37)*	0.046*** *(5.69)*
Board	−0.356*** *(-4.54)*	−0.344*** *(-4.42)*	−0.367*** *(-4.94)*	−0.350*** *(-4.80)*	−0.359*** *(-4.52)*	−0.331*** *(-4.20)*	−0.351*** *(-4.52)*	−0.335*** *(-3.79)*	−0.344*** *(-4.85)*	−0.342*** *(-4.28)*	−0.370*** *(-4.83)*	−0.353*** *(-4.36)*
M/B Ratio	0.002 *(0.89)*	0.002 *(0.74)*	0.002 *(1.07)*	0.002 *(0.89)*	0.001 *(0.71)*	0.002 *(1.11)*	0.002 *(0.82)*	0.001 *(0.60)*	0.001 *(0.77)*	0.003 *(1.33)*	0.002 *(1.02)*	0.002 *(0.78)*
ROA		0.188*** *(3.63)*		0.167*** *(3.38)*		0.142*** *(2.73)*		0.162*** *(2.77)*		0.177*** *(2.63)*		0.171*** *(3.22)*
EPS		−0.053** *(-1.98)*		−0.037 *(-1.51)*		−0.014 *(-0.54)*		−0.035 *(-1.15)*		−0.021 *(-0.78)*		−0.029 *(-1.05)*
Rep Tax			0.488 *(1.63)*	0.511* *(1.71)*					0.546 *(1.21)*	−0.466 *(-0.73)*		
Div Tax					−1.182*** *(-2.98)*	−1.148*** *(-2.93)*			−0.806 *(-1.36)*	−2.082** *(-2.54)*		
Tax Interaction							−1.467 *(-1.54)*	−1.133 *(-1.05)*	−1.061 *(-0.65)*	2.730 *(1.23)*		
Tax Friendly											−0.095* *(-1.76)*	−0.089 *(-1.61)*
Constant	−0.270*** *(-2.63)*	−0.252** *(-2.48)*	−0.422*** *(-2.60)*	−0.430*** *(-2.69)*	0.138 *(1.05)*	0.104 *(0.81)*	−0.109 *(-0.81)*	−0.137 *(-0.88)*	−0.062 *(-0.30)*	0.275 *(1.02)*	−0.223** *(-2.23)*	−0.211** *(-2.01)*
Pseudo R^2^	0.085	0.091	0.088	0.095	0.093	0.101	0.087	0.092	0.099	0.103	0.087	0.094

Panel II: Medium Sized												
	I	II	III	IV	V	VI	VII	VIII	IX	X	XI	XII
Debt Ratio	−0.008 *(-1.62)*	−0.009* *(-1.68)*	−0.008* *(-1.67)*	−0.009* *(-1.71)*	−0.009 *(-1.61)*	−0.009 *(-1.62)*	−0.008 *(-1.61)*	−0.012** *(-2.04)*	−0.008 *(-1.47)*	−0.009 *(-1.60)*	−0.008 *(-1.58)*	−0.009 *(-1.58)*
Firm Size	0.029*** *(4.18)*	0.031*** *(4.14)*	0.027*** *(4.06)*	0.032*** *(4.19)*	0.030*** *(4.05)*	0.033*** *(4.16)*	0.028*** *(4.07)*	0.031*** *(3.95)*	0.029*** *(3.72)*	0.031*** *(3.98)*	0.029*** *(4.02)*	0.031*** *(3.87)*
Board	−0.543*** *(-7.62)*	−0.434*** *(-5.70)*	−0.460*** *(-6.51)*	−0.433*** *(-5.60)*	−0.485*** *(-6.25)*	−0.416*** *(-5.05)*	−0.520*** *(-7.33)*	−0.434*** *(-5.48)*	−0.425*** *(-5.25)*	−0.383*** *(-4.74)*	−0.543*** *(-7.32)*	−0.434*** *(-5.32)*
M/B Ratio	0.002 *(1.00)*	0.002 *(1.07)*	0.002 *(1.07)*	0.002 *(1.07)*	0.002 *(0.98)*	0.002 *(0.99)*	0.002 *(1.01)*	0.003 *(1.18)*	0.002 *(0.90)*	0.002 *(1.02)*	0.002 *(0.97)*	0.002 *(1.01)*
ROA		0.042 *(0.84)*		0.043 *(0.82)*		0.084 *(1.55)*		0.044 *(0.83)*		0.073 *(1.07)*		0.042 *(0.80)*
EPS		−0.049* *(-1.87)*		−0.049* *(-1.86)*		−0.044 *(-1.58)*		−0.044 *(-1.60)*		−0.045 *(-1.62)*		−0.049* *(-1.76)*
Rep Tax			0.353 *(1.24)*	0.465 *(1.47)*					0.545 *(1.06)*	0.266 *(0.41)*		
Div Tax					−0.556 *(-1.43)*	−0.731* *(-1.78)*			−0.266 *(-0.39)*	−0.857 *(-1.03)*		
Tax Interaction							−0.747 *(-0.86)*	−0.798 *(-0.83)*	−1.041 *(-0.56)*	0.331 *(0.15)*		
Tax Friendly											0.013 *(0.27)*	−0.001 *(-0.02)*
Constant	0.235** *(2.52)*	0.181* *(1.83)*	0.082 *(0.54)*	−0.014 *(-0.08)*	0.338*** *(2.64)*	0.321** *(2.36)*	0.313** *(2.55)*	0.262* *(1.89)*	0.141 *(0.60)*	0.227 *(0.83)*	0.235** *(2.42)*	0.181* *(1.71)*
Pseudo R^2^	0.047	0.054	0.049	0.055	0.049	0.057	0.048	0.055	0.052	0.058	0.047	0.054

Panel III: Large Sized												
	I	II	III	IV	V	VI	VII	VIII	IX	X	XI	XII
Debt Ratio	−0.003 *(-0.32)*	−0.005 *(-0.52)*	−0.005 *(-0.45)*	−0.005 *(-0.50)*	−0.0004 *(-0.05)*	−0.004 *(-0.42)*	−0.0004 *(-0.04)*	−0.002 *(-0.21)*	−0.001 *(-0.15)*	−0.002 *(-0.20)*	−0.002 *(-0.26)*	−0.003 *(-0.29)*
Firm Size	0.018 *(1.34)*	0.027** *(1.99)*	0.025* *(1.68)*	0.027* *(1.92)*	0.017 *(1.27)*	0.024* *(1.88)*	0.017 *(1.15)*	0.023* *(1.68)*	0.021 *(1.37)*	0.023 *(1.64)*	0.016 *(1.13)*	0.019 *(1.28)*
Board	−0.071 *(-0.50)*	0.018 *(0.13)*	−0.076 *(-0.50)*	0.018 *(0.13)*	−0.139 *(-0.97)*	0.017 *(0.13)*	−0.139 *(-0.89)*	0.134 *(0.95)*	−0.069 *(-0.43)*	0.134 *(0.91)*	−0.082 *(-0.55)*	0.065 *(0.43)*
M/B Ratio	0.003 *(0.63)*	0.003 *(0.75)*	0.003 *(0.63)*	0.003 *(0.72)*	0.002 *(0.51)*	0.003 *(0.71)*	0.002 *(0.46)*	0.002 *(0.60)*	0.002 *(0.50)*	0.002 *(0.59)*	0.003 *(0.59)*	0.003 *(0.63)*
ROA		0.144 *(1.58)*		0.144 *(1.49)*		0.143 *(1.55)*		0.153 *(1.63)*		0.153 *(1.23)*		0.149 *(1.50)*
EPS		−0.101** *(-2.13)*		−0.101** *(-2.07)*		−0.099** *(-2.09)*		−0.099** *(-2.00)*		−0.099* *(-1.93)*		−0.107** *(-2.07)*
Rep Tax			0.547 *(0.88)*	0.000 *(0.00)*					1.070 *(1.04)*	0.203 *(0.17)*		
Div Tax					−0.504 *(-0.70)*	−0.212 *(-0.31)*			0.371 *(0.27)*	−0.325 *(-0.22)*		
Tax Interaction							−1.260 *(-0.65)*	−1.759 *(-1.02)*	−2.735 *(-0.74)*	−0.944 *(-0.23)*		
Tax Friendly											0.077 *(0.74)*	0.099 *(0.95)*
Constant	0.321* *(1.71)*	0.186 *(1.04)*	0.028 *(0.09)*	0.186 *(0.60)*	0.475** *(2.00)*	0.258 *(1.12)*	0.475* *(1.74)*	0.339 *(1.36)*	0.038 *(0.08)*	0.257 *(0.52)*	0.353* *(1.80)*	0.259 *(1.32)*
Pseudo R^2^	0.003	0.019	0.005	0.019	0.005	0.019	0.004	0.020	0.008	0.020	0.004	0.021

Superscripts state the level of statistical significance = 1 % (***), 5 % (**) and 10 % (*), t-statistics are stated in the parenthesis.

Superscripts state the level of statistical significance = 1 % (***), 5 % (**) and 10 % (*), t-statistics are stated in the parenthesis.

Superscripts state the level of statistical significance = 1 % (***), 5 % (**) and 10 % (*), t-statistics are stated in the parenthesis.

**Table 6:** Determinants of repurchase financing: Aggregate.

	I	II	III	IV	V	VI	VII	VIII	IX	X	XI	XII
Debt Ratio	0.464*** *(5.50)*	0.475*** *(5.55)*	0.455*** *(5.40)*	0.466*** *(5.46)*	0.466*** *(5.53)*	0.477*** *(5.60)*	0.464*** *(5.49)*	0.475*** *(5.55)*	0.456*** *(5.41)*	0.468*** *(5.48)*	0.455*** *(5.39)*	0.465*** *(5.44)*
Firm Size	0.040 *(0.36)*	0.021 *(0.19)*	0.067 *(0.60)*	0.045 *(0.39)*	0.054 *(0.49)*	0.032 *(0.28)*	0.040 *(0.36)*	0.021 *(0.19)*	0.073 *(0.66)*	0.048 *(0.42)*	0.068 *(0.61)*	0.047 *(0.40)*
Board	−0.172 *(-0.15)*	0.177 *(0.15)*	0.130 *(0.11)*	0.386 *(0.33)*	0.065 *(0.06)*	0.533 *(0.45)*	−0.179 *(-0.16)*	0.183 *(0.16)*	0.308 *(0.26)*	0.565 *(0.47)*	0.066 *(0.06)*	0.410 *(0.35)*
M/B Ratio	−0.118*** *(-3.00)*	−0.119*** *(-2.96)*	−0.115*** *(-2.93)*	−0.117*** *(-2.92)*	−0.120*** *(-3.05)*	−0.120*** *(-3.01)*	−0.118*** *(-3.00)*	−0.119*** *(-2.96)*	−0.114*** *(-2.89)*	−0.117*** *(-2.94)*	−0.116*** *(-2.97)*	−0.117*** *(-2.93)*
ROA		2.207 *(1.25)*		1.935 *(1.11)*		2.199 *(1.39)*		2.207 *(1.25)*		1.801 *(1.02)*		2.073 *(1.25)*
EPS		0.164 *(0.38)*		0.188 *(0.44)*		0.268 *(0.62)*		0.168 *(0.39)*		0.279 *(0.64)*		0.211 *(0.49)*
Rep Tax			8.936* *(1.84)*	8.080 *(1.60)*					26.693 *(0.53)*	26.221 *(0.46)*		
Div Tax					−6.598 *(-1.15)*	−8.960 *(-1.49)*			20.513 *(0.31)*	19.555 *(0.26)*		
Tax Interaction							1.070 *(0.08)*	−0.851 *(-0.06)*	−66.911 *(-0.40)*	−67.171 *(-0.35)*		
Tax Friendly											−1.215 *(-1.50)*	−1.241 *(-1.52)*
Constant	0.714 *(0.49)*	0.515 *(0.34)*	−3.188 *(-1.24)*	−2.957 *(-1.11)*	2.066 *(1.10)*	2.413 *(1.22)*	0.615 *(0.32)*	0.596 *(0.29)*	−8.923 *(-0.45)*	−8.557 *(-0.38)*	0.379 *(0.26)*	0.220 *(0.14)*
Pseudo R^2^	0.018	0.019	0.019	0.021	0.018	0.020	0.018	0.019	0.020	0.021	0.019	0.021

Superscripts state the level of statistical significance = 1 % (***), 5 % (**) and 10 % (*), t-statistics are stated in the parenthesis.

**Table 7:** Determinants of repurchase financing: Size-specific.

Panel I: Small Sized												
	I	II	III	IV	V	VI	VII	VIII	IX	X	XI	XII
Debt Ratio	0.092*** *(4.17)*	0.127*** *(5.53)*	0.092*** *(4.33)*	0.136*** *(6.59)*	0.082*** *(3.88)*	0.084*** *(3.60)*	0.098*** *(4.47)*	0.128*** *(5.79)*	0.095*** *(4.38)*	0.144*** *(6.94)*	0.105*** *(4.92)*	0.136*** *(6.36)*
Firm Size	0.049* *(1.75)*	0.041 *(1.32)*	0.053* *(1.94)*	0.039 *(1.42)*	0.045* *(1.65)*	0.044 *(1.41)*	0.050* *(1.77)*	0.040 *(1.36)*	0.051* *(1.81)*	0.039 *(1.41)*	0.048* *(1.73)*	0.038 *(1.33)*
Board	−0.365 *(-1.25)*	−0.341 *(-1.10)*	−0.377 *(-1.33)*	−0.331 *(-1.18)*	−0.373 *(-1.31)*	−0.285 *(-0.88)*	−0.423 *(-1.47)*	−0.337 *(-1.13)*	−0.389 *(-1.32)*	−0.305 *(-1.06)*	−0.316 *(-1.12)*	−0.355 *(-1.22)*
M/B Ratio	−0.022** *(-2.14)*	−0.028*** *(-2.64)*	−0.022** *(-2.22)*	−0.030*** *(-3.11)*	−0.020** *(-2.06)*	−0.021* *(-1.90)*	−0.023** *(-2.27)*	−0.028*** *(-2.76)*	−0.022** *(-2.20)*	−0.031*** *(-3.23)*	−0.024** *(-2.44)*	−0.030*** *(-3.00)*
ROA		0.457** *(2.21)*		0.399** *(210)*		0.396* *(1.85)*		0.471** *(2.36)*		0.457* *(1.89)*		0.365* *(1.91)*
EPS		0.039 *(0.37)*		0.103 *(1.07)*		0.051 *(0.47)*		0.030 *(0.38)*		0.146 *(1.46)*		0.121 *(1.22)*
Rep Tax			1.971* *(1.72)*	2.280** *(1.98)*					0.557 *(0.30)*	−1.811 *(-0.79)*		
Div Tax					−0.562 *(-0.40)*	−0.772 *(-0.48)*			−2.266 *(-0.92)*	−7.447** *(-2.53)*		
Tax Interaction							0.898 *(0.25)*	0.164 *(0.05)*	5.096 *(0.76)*	19.513** *(2.45)*		
Tax Friendly											−0.253 *(-1.27)*	−0.339* *(-1.69)*
Constant	−0.179 *(-0.47)*	−0.190 *(-0.47)*	−0.998 *(-1.61)*	−1.089* *(-1.77)*	−0.007 *(-0.02)*	−0.056 *(-0.11)*	−0.259 *(-0.51)*	−0.205 *(-0.39)*	−0.336 *(-0.39)*	0.442 *(0.46)*	−0.170 *(-0.46)*	−0.147 *(-0.39)*
Pseudo R^2^	0.010	0.011	0.011	0.012	0.010	0.011	0.010	0.011	0.012	0.013	0.011	0.012

Panel II: Medium Sized												
	I	II	III	IV	V	VI	VII	VIII	IX	X	XI	XII
Debt Ratio	0.409*** *(18.66)*	0.407*** *(17.81)*	0.404*** *(17.77)*	0.404*** *(17.60)*	0.406*** *(19.16)*	0.407*** *(17.97)*	0.408*** *(17.73)*	0.407*** *(17.42)*	0.405*** *(18.26)*	0.418*** *(18.61)*	0.403*** *(18.40)*	0.404*** *(17.27)*
Firm Size	−0.009 *(-0.34)*	−0.008 *(-0.28)*	0.004 *(0.15)*	0.003 *(0.10)*	0.002 *(0.08)*	−0.003 *(-0.13)*	−0.008 *(-0.28)*	−0.007 *(-0.25)*	0.010 *(0.37)*	0.005 *(0.18)*	0.008 *(0.29)*	−0.0004 *(-0.01)*
Board	−0.292 *(-1.01)*	−0.270 *(-0.88)*	−0.216 *(-0.71)*	−0.243 *(-0.78)*	−0.226 *(-0.80)*	−0.213 *(-0.68)*	−0.314 *(-1.03)*	−0.281 *(-0.89)*	−0.201 *(-0.67)*	−0.233 *(-0.75)*	−0.180 *(-0.62)*	−0.242 *(-0.76)*
M/B Ratio	−0.074*** *(-7.29)*	−0.074*** *(-6.90)*	−0.074*** *(-6.97)*	−0.073*** *(-6.86)*	−0.074*** *(-7.52)*	−0.074*** *(-6.99)*	−0.074*** *(-6.93)*	−0.074*** *(-6.76)*	−0.075*** *(-7.24)*	−0.076*** *(-7.25)*	−0.074*** *(-7.26)*	−0.074*** *(-6.75)*
ROA		−0.119 *(-0.58)*		−0.119 *(-0.56)*		−0.118 *(-0.58)*		−0.118 *(-0.56)*		−0.130 *(-0.50)*		−0.126 *(-0.60)*
EPS		−0.004 *(-0.04)*		0.005 *(0.05)*		0.005 *(0.05)*		−0.015 *(-0.14)*		0.028 *(0.26)*		0.060 *(0.56)*
Rep Tax			1.007 *(0.82)*	0.977 *(0.77)*					−0.429 *(-0.22)*	0.496 *(0.20)*		
Div Tax					−1.239 *(-0.88)*	−1.176 *(-0.76)*			−2.786 *(-0.11)*	−2.145 *(-0.68)*		
Tax Interaction							0.218 *(0.06)*	0.221 *(0.06)*	4.832 *(0.70)*	3.092 *(0.36)*		
Tax Friendly											−0.243 *(-1.18)*	−0.308 *(-1.40)*
Constant	0.825** *(2.18)*	0.825** *(2.05)*	0.212 *(0.32)*	0.265 *(0.39)*	0.944** *(2.01)*	1.013** *(1.97)*	0.799 *(1.52)*	0.803 *(1.45)*	0.920 *(1.05)*	0.650 *(0.62)*	0.551 *(1.45)*	0.683 *(1.65)*
Pseudo R^2^	0.040	0.041	0.041	0.042	0.041	0.042	0.040	0.041	0.042	0.042	0.041	0.042

Panel III: Large Sized												
	I	II	III	IV	V	VI	VII	VIII	IX	X	XI	XII
Debt Ratio	0.658*** *(11.20)*	0.714*** *(13.70)*	0.658*** *(11.33)*	0.714*** *(13.57)*	0.689*** *(12.18)*	0.713*** *(13.11)*	0.693*** *(11.50)*	0.713*** *(12.99)*	0.723*** *(11.86)*	0.708*** *(13.84)*	0.665*** *(11.48)*	0.714*** *(13.98)*
Firm Size	−0.026 *(-0.35)*	0.003 *(0.05)*	−0.026 *(-0.35)*	0.002 *(0.04)*	−0.030 *(-0.41)*	0.004 *(0.06)*	−0.032 *(-0.41)*	0.004 *(0.06)*	−0.020 *(-0.26)*	−0.002 *(-0.04)*	−0.027 *(-0.36)*	0.002 *(0.04)*
Board	0.052 *(0.07)*	−0.071 *(-0.10)*	0.052 *(0.07)*	−0.067 *(-0.09)*	0.067 *(0.09)*	−0.056 *(-0.08)*	0.081 *(0.10)*	−0.065 *(-0.09)*	0.009 *(0.01)*	0.052 *(0.08)*	0.053 *(0.07)*	−0.065 *(-0.09)*
M/B Ratio	−0.119*** *(-4.37)*	−0.147*** *(-6.02)*	−0.119*** *(-4.42)*	−0.147*** *(-5.96)*	−0.120*** *(-4.56)*	−0.143*** *(-5.62)*	−0.122*** *(-4.31)*	−0.145*** *(-5.66)*	−0.128*** *(-4.50)*	−0.139*** *(-5.78)*	−0.120*** *(-4.46)*	−0.147*** *(-6.14)*
ROA		−0.344 *(-0.74)*		−0.350 *(-0.72)*		−0.347 *(-0.70)*		−0.338 *(-0.69)*		−1.092* *(-1.83)*		−0.344 *(-0.75)*
EPS		−0.177 *(-0.73)*		−0.173 *(-0.71)*		−0.162 *(-0.64)*		−0.179 *(-0.69)*		−0.154 *(-0.63)*		−0.168 *(-0.71)*
Rep Tax			−0.751 *(-0.24)*	0.081 *(0.03)*					−1.366 *(-0.26)*	4.809 *(0.86)*		
Div Tax					−2.148 *(-0.57)*	0.266 *(0.07)*			−2.857 *(-0.41)*	6.261 *(0.87)*		
Tax Interaction							1.148 *(0.12)*	0.318 *(0.04)*	4.093 *(0.22)*	−16.072 *(-0.82)*		
Tax Friendly											−0.039 *(-0.07)*	0.049 *(0.10)*
Constant	1.278 *(1.26)*	1.117 *(1.22)*	1.578 *(0.93)*	1.091 *(0.70)*	1.854 *(1.48)*	1.019 *(0.83)*	1.219 *(0.88)*	1.062 *(0.82)*	2.095 *(0.87)*	−0.684 *(-0.29)*	1.292 *(1.28)*	1.121 *(1.24)*
Pseudo R^2^	0.110	0.115	0.110	0.115	0.110	0.115	0.110	0.115	0.111	0.116	0.110	0.115

Superscripts state the level of statistical significance = 1 % (***), 5 % (**) and 10 % (*), t-statistics are stated in the parenthesis.

Superscripts state the level of statistical significance = 1 % (***), 5 % (**) and 10 % (*), t-statistics are stated in the parenthesis.

Superscripts state the level of statistical significance = 1 % (***), 5 % (**) and 10 % (*), t-statistics are stated in the parenthesis.

From the size-specific testing, small sized dividends are positively influenced by *Firm Size*, *ROA* and mildly by *Rep Tax*, and negatively influenced by *Debt Ratio*, *Board*, Div *Tax*, and mildly by *EPS* and *Tax Friendly*. As we progress to those that are of medium sized, we see that the influences narrow, since all but *ROA* and *Rep Tax* sustain their significance with *Debt Ratio* losing some strength. Further narrowing is seen with those that are of large size, as only *EPS* maintains its significance and strength, followed by *Firm Size* which retains its significance but loses strength.

Thus, dividends of all sizes are intended to offset the impact of reduced earnings by signalling future strength, which is aligned with the positive effect of having larger asset holdings, but the influence of generating good returns against these assets only boosts small sized dividends. Lower leveraged firms are likely to pass on the cost savings from debt servicing via small and medium sized dividends, while these are further supported when dividends are subjected to lower tax and are tax efficient than repurchases, but also continue to be restricted by independent directors. Further, when taxation on repurchases increases small sized dividends are limitedly seen as their replacements.

Small sized repurchases are positively influenced by *Debt Ratio*, *ROA* and mildly by *Firm Size*, *Rep Tax* and *Tax Interaction*, and negatively influenced by *M/B Ratio*, and mildly by Div *Tax* and *Tax Friendly*. Here too we see a narrowing of influences upon size increment, but this is rather magnified. Medium and large sized repurchases are subject to identical results, which are the positive influence of *Debt Ratio* and negative influence of *M/B Ratio*. Thus, the pre-existing high debt appetite continues to promote repurchases of all sizes, while they are being continually used for signalling stock undervaluation. Further, if a firm has a strong asset base and generates good returns against these assets, then small repurchases are more likely. These are also maximised to offset the effect of higher tax on repurchases, and dividends’ tax efficiency over repurchases.

Given these findings, we accept the null hypothesis H2_0_, and the alternative hypotheses, H3_1_, H4_1_, H5_1_, H6_1_, H7_1_, H8_1_, H9_1_, and H10_1_.

### 4.2 Robustness testing

In Table 8, Table 9 we tabulate the results from the testing of aggregate (Equation (5)) and size-specific (Equation (6)) total payouts, respectively. For the aggregate results we see a combination of influences witnessed for aggregate dividends and repurchases, however the latter is more dominant. The positive influence of *Firm Size* and negative influence of *Board* are consistent with the findings of aggregate dividends, while the negative influence of *Debt Ratio* and positive influences of *M/B Ratio* and *Rep Tax* are consistent with the findings of aggregate repurchases. The insignificance of *ROA* and *EPS* is inconsistent with the positive and negative influences, respectively, seen with aggregate dividends, but remain consistent with the findings of aggregate repurchases. With small sized payouts the initial results indicated homogeneity between the determinants of dividends and repurchases, and concurrently we see that all of the influences seen with the two payouts reappear, except for *EPS’* insignificance which showed a very mild negative influence for the dividend testing.

**Table 8:** Determinants of total payout financing: Aggregate.

	I	II	III	IV	V	VI	VII	VIII	IX	X	XI	XII
Debt Ratio	−0.142*** *(-8.90)*	−0.142*** *(-8.92)*	−0.144*** *(-9.05)*	−0.144*** *(-9.04)*	−0.143*** *(-8.92)*	−0.143*** *(-8.92)*	−0.143*** *(-8.98)*	−0.144*** *(-9.00)*	−0.145*** *(-9.10)*	−0.145*** *(-9.09)*	−0.144*** *(-9.02)*	−0.144*** *(-9.04)*
Firm Size	1.015*** *(48.92)*	1.014*** *(46.95)*	1.020*** *(49.08)*	1.018*** *(47.03)*	1.014*** *(48.59)*	1.013*** *(46.83)*	1.015*** *(49.06)*	1.015*** *(47.08)*	1.018*** *(48.86)*	1.017*** *(49.69)*	1.020*** *(48.74)*	1.019*** *(46.88)*
Board	−0.611*** *(-2.89)*	−0.570*** *(-2.64)*	−0.539** *(-2.53)*	−0.521** *(-2.41)*	−0.631*** *(-2.94)*	−0.587*** *(-2.66)*	−0.630*** *(-2.98)*	−0.590*** *(-2.74)*	−0.558*** *(-2.58)*	−0.558** *(-2.53)*	−0.558*** *(-2.62)*	−0.515** *(-2.37)*
M/B Ratio	0.038*** *(5.16)*	0.039*** *(5.23)*	0.039*** *(5.29)*	0.039*** *(5.31)*	0.038*** *(5.19)*	0.039*** *(5.24)*	0.038*** *(5.20)*	0.039*** *(5.28)*	0.040*** *(5.37)*	0.040*** *(5.36)*	0.038*** *(5.20)*	0.039*** *(5.28)*
ROA		0.125 *(0.87)*		0.066 *(0.45)*		0.117 *(0.80)*		0.131 *(0.91)*		−0.006 *(-0.03)*		0.128 *(0.90)*
Earnings		0.004 *(0.06)*		0.006 *(0.09)*		0.001 *(0.02)*		−0.012 *(-0.17)*		0.006 *(0.08)*		0.007 *(0.11)*
Rep. Tax			1.655* *(1.92)*	1.555* *(1.75)*					2.317* *(1.68)*	2.352 *(1.35)*		
Div. Tax					0.581 *(0.54)*	0.399 *(0.36)*			1.711 *(0.95)*	1.749 *(0.77)*		
Tax Interaction							3.574 *(1.38)*	3.609 *(1.37)*	−2.257 *(-0.46)*	−2.397 *(-0.39)*		
Tax Friendly											−0.241 *(-1.60)*	−0.244 *(-1.63)*
Constant	−2.048*** *(-7.40)*	−2.062*** *(-7.31)*	−2.770*** *(-5.94)*	−2.732*** *(-5.76)*	−2.168*** *(-6.11)*	−2.146*** *(-5.90)*	−2.381*** *(-6.49)*	−2.411*** *(-6.36)*	−3.204*** *(-5.06)*	−3.209*** *(-4.31)*	−2.112*** *(-7.58)*	−2.126*** *(-7.49)*
Pseudo R^2^	0.439	0.439	0.441	0.441	0.439	0.439	0.440	0.441	0.442	0.442	0.440	0.441

Superscripts state the level of statistical significance = 1 % (***), 5 % (**) and 10 % (*), t-statistics are stated in the parenthesis.

**Table 9:** Determinants of total payout financing: Size-specific.

Panel I: Small Sized												
	I	II	III	IV	V	VI	VII	VIII	IX	X	XI	XII
Debt Ratio	−0.132*** *(-5.34)*	−0.139*** *(-5.91)*	−0.135*** *(-5.41)*	−0.141*** *(-5.89)*	−0.140*** *(-5.82)*	−0.140*** *(-5.70)*	−0.139*** *(-5.63)*	−0.143*** *(-5.92)*	−0.138*** *(-5.51)*	−0.134*** *(-5.72)*	−0.133*** *(-4.86)*	−0.141*** *(-5.49)*
Firm Size	1.035*** *(32.24)*	1.028*** *(32.45)*	1.054*** *(32.27)*	1.039*** *(31.85)*	1.029*** *(32.88)*	1.027*** *(30.87)*	1.029*** *(32.13)*	1.030*** *(31.66)*	1.029*** *(31.45)*	1.024*** *(32.27)*	1.042*** *(28.93)*	1.044*** *(29.77)*
Board	−0.794** *(-2.43)*	−0.763** *(-2.41)*	−0.805** *(-2.40)*	−0.784** *(-2.40)*	−0.108*** *(-3.17)*	−0.727** *(-2.14)*	−0.969*** *(-2.96)*	−0.866*** *(-2.66)*	−0.778** *(-2.29)*	−0.708** *(-2.20)*	−0.777** *(-2.12)*	−0.735** *(-2.10)*
M/B Ratio	0.115*** *(9.94)*	0.117*** *(10.60)*	0.110*** *(9.38)*	0.115*** *(10.18)*	0.108*** *(9.62)*	0.115*** *(9.93)*	0.108*** *(9.41)*	0.110*** *(9.74)*	0.112*** *(9.55)*	0.105*** *(9.60)*	0.112*** *(8.79)*	0.113*** *(9.37)*
ROA		0.403* *(1.91)*		0.259 *(1.33)*		0.371* *(1.66)*		0.376* *(1.74)*		1.188*** *(4.36)*		0.394* *(1.70)*
EPS		0.091 *(0.83)*		0.098 *(0.88)*		0.083 *(0.72)*		0.075 *(0.66)*		0.006 *(0.05)*		0.089 *(0.74)*
Rep Tax			3.785*** *(2.79)*	1.066 *(0.80)*					2.828 *(1.31)*	−4.184 *(-1.63)*		
Div Tax					2.895* *(1.80)*	0.621 *(0.37)*			2.450 *(0.86)*	−7.192** *(-2.17)*		
Tax Interaction							6.971* *(1.74)*	4.572 *(1.15)*	−1.523 *(-0.20)*	21.479** *(2.41)*		
Tax Friendly											−0.254 *(-0.98)*	−0.373 *(-1.54)*
Constant	−2.927*** *(-6.84)*	−2.881*** *(-6.96)*	−4.639 *(-6.32)*	−3.413*** *(-4.78)*	−3.379*** *(-6.35)*	−2.999*** *(-5.36)*	−3.387*** *(-5.97)*	−3.257*** *(-5.70)*	−4.396*** *(-4.42)*	−1.478 *(-1.36)*	−2.995*** *(-6.25)*	−3.063*** *(-6.69)*
Pseudo R^2^	0.641	0.648	0.645	0.649	0.644	0.649	0.644	0.650	0.648	0.652	0.643	0.650

Panel II: Medium Sized												
	I	II	III	IV	V	VI	VII	VIII	IX	X	XI	XII
Debt Ratio	−0.161*** *(-9.81)*	−0.154*** *(-9.37)*	−0.161*** *(-9.41)*	−0.155*** *(-9.26)*	−0.165*** *(-10.38)*	−0.156*** *(-9.88)*	−0.167*** *(-10.26)*	−0.154*** *(-9.59)*	−0.166*** *(-10.35)*	−0.159*** *(-9.45)*	−0.161*** *(-10.04)*	−0.155*** *(-9.07)*
Firm Size	1.010*** *(47.38)*	1.017*** *(45.77)*	1.011*** *(45.22)*	1.017*** *(44.74)*	1.008*** *(48.68)*	1.011*** *(47.39)*	1.016*** *(48.23)*	1.015*** *(46.70)*	1.010*** *(48.18)*	1.012*** *(44.39)*	1.011*** *(48.01)*	1.017*** *(43.83)*
Board	−0.458** *(-2.11)*	−0.288 *(-1.30)*	−0.462** *(-2.01)*	−0.320 *(-1.41)*	−0.451** *(-2.12)*	−0.390* *(-1.79)*	−0.472** *(-2.20)*	−0.347 *(-1.60)*	−0.454** *(-2.09)*	−0.375 *(-1.62)*	−0.468** *(-2.18)*	−0.314 *(-1.35)*
M/B Ratio	0.142*** *(18.53)*	0.140*** *(18.17)*	0.141*** *(17.58)*	0.140*** *(17.81)*	0.138*** *(18.69)*	0.129*** *(17.36)*	0.134*** *(17.64)*	0.137*** *(18.20)*	0.143*** *(18.91)*	0.143*** *(18.07)*	0.141*** *(18.82)*	0.139*** *(17.38)*
ROA		0.720*** *(4.86)*		0.676*** *(4.38)*		0.523*** *(3.64)*		0.708*** *(4.90)*		0.328* *(1.68)*		0.713*** *(4.66)*
EPS		−0.058 *(-0.77)*		−0.056 *(-0.72)*		−0.062 *(-0.85)*		−0.080 *(-1.06)*		−0.053 *(-0.66)*		−0.058 *(-0.74)*
Rep Tax			0.466 *(0.50)*	0.435 *(0.47)*					3.794*** *(2.73)*	1.948 *(1.06)*		
Div Tax					2.683** *(2.53)*	2.482** *(2.29)*			6.337*** *(3.48)*	3.748 *(1.58)*		
Tax Interaction							5.110* *(1.94)*	5.437** *(2.06)*	−8.964* *(-1.80)*	−3.083 *(-0.48)*		
Tax Friendly											−0.071 *(-0.47)*	−0.094 *(-0.59)*
Constant	−2.112*** *(-7.43)*	−2.346*** *(-8.08)*	−2.299*** *(-4.58)*	−2.480*** *(-4.98)*	−2.757*** *(-7.84)*	−2.798*** *(-7.80)*	−2.650*** *(-7.10)*	−2.796*** *(-7.33)*	−4.315*** *(-6.76)*	−3.605*** *(-4.60)*	−2.112*** *(-7.54)*	−2.312*** *(-7.63)*
Pseudo R^2^	0.696	0.701	0.697	0.701	0.699	0.704	0.699	0.703	0.704	0.705	0.697	0.701

Panel III: Large Sized												
	I	II	III	IV	V	VI	VII	VIII	IX	X	XI	XII
Debt Ratio	−0.140*** *(-8.27)*	−0.139*** *(-8.66)*	−0.139*** *(-8.29)*	−0.135*** *(-8.59)*	−0.140*** *(-7.97)*	−0.141*** *(-8.36)*	−0.141*** *(-8.39)*	−0.138*** *(-7.76)*	−0.141*** *(-8.18)*	−0.137*** *(-8.24)*	−0.140*** *(-8.18)*	−0.139*** *(-8.91)*
Firm Size	0.975*** *(44.38)*	0.980*** *(45.20)*	0.971*** *(44.28)*	0.976*** *(45.77)*	0.971*** *(42.38)*	0.984*** *(43.15)*	0.973*** *(44.79)*	0.981*** *(40.88)*	0.972*** *(43.23)*	0.960*** *(42.55)*	0.972*** *(43.44)*	0.977*** *(45.90)*
Board	−0.225 *(-1.01)*	−0.114 *(-0.53)*	−0.198 *(-0.88)*	−0.091 *(-0.43)*	−0.269 *(-1.14)*	−0.099 *(-0.43)*	−0.231 *(-1.05)*	−0.092 *(-0.38)*	−0.171 *(-0.73)*	−0.117 *(-0.51)*	−0.196 *(-0.86)*	−0.097 *(-0.46)*
M/B Ratio	0.136*** *(17.18)*	0.125*** *(16.53)*	0.134*** *(17.22)*	0.127*** *(17.21)*	0.138*** *(16.77)*	0.133*** *(16.79)*	0.136*** *(17.38)*	0.131*** *(15.70)*	0.137*** *(16.90)*	0.133*** *(16.98)*	0.134*** *(16.92)*	0.125*** *(17.06)*
ROA		0.508*** *(3.52)*		0.432*** *(2.98)*		0.571*** *(3.72)*		0.463*** *(2.90)*		0.113 *(0.59)*		0.411*** *(2.93)*
EPS		−0.018 *(-0.25)*		−0.007 *(-0.11)*		−0.032 *(-0.41)*		−0.016 *(-0.20)*		0.073 *(0.92)*		−0.0008 *(-0.01)*
Rep Tax			0.439 *(0.48)*	0.300 *(0.34)*					4.838*** *(3.25)*	4.156** *(2.28)*		
Div Tax					0.777 *(0.66)*	0.670 *(0.58)*			6.289*** *(3.22)*	5.101** *(2.17)*		
Tax Interaction							1.640 *(0.60)*	1.184 *(0.41)*	−14.357*** *(-2.69)*	−11.956* *(-1.88)*		
Tax Friendly											−0.095 *(-0.59)*	−0.068 *(-0.46)*
Constant	−1.440*** *(-4.92)*	−1.573*** *(-5.56)*	−1.568*** *(-3.18)*	−1.662*** *(-3.56)*	−1.583*** *(-4.06)*	−1.830*** *(-4.77)*	−1.579*** *(-4.10)*	−1.729*** *(-4.10)*	−3.498*** *(-5.12)*	−3.072*** *(-3.97)*	−1.397*** *(-4.69)*	−1.531*** *(-5.51)*
Pseudo R^2^	0.711	0.713	0.711	0.714	0.711	0.714	0.711	0.714	0.715	0.716	0.711	0.714

Superscripts state the level of statistical significance = 1 % (***), 5 % (**) and 10 % (*), t-statistics are stated in the parenthesis.

Superscripts state the level of statistical significance = 1 % (***), 5 % (**) and 10 % (*), t-statistics are stated in the parenthesis.

Superscripts state the level of statistical significance = 1 % (***), 5 % (**) and 10 % (*), t-statistics are stated in the parenthesis.

In the case of medium sized payouts, we find an interesting pattern. Firstly, we continue to see all of the significant determinants from repurchase testing reappearing, while those related to dividends not being fully represented, which is seen with *EPS’* insignificance instead of being negative. Secondly, we see significance of new variables that were not see during the initial dividend and repurchase testing, these include the positive influences of *ROA* and *Rep Tax*, and positive-to-negative influence of *Tax Interaction*. Thus, the aggregate corporate payout policy for medium sized payouts is also determined by how efficiently the firm's assets are being employed for profit generation, and the tax framework. The fluctuating influence of *Tax Interaction* is attributable to the UK seeing a rather variation in the tax rates (as discussed in Section 2). Hence, the co-movement of tax rates or the movement of one of the tax rates with the other remaining constant will result in a bi-directional influence on corporate payout policy. Large sized payouts also follow a similar pattern. All of the influences seen with large sized dividends and repurchases reappear, except former's *EPS’* negative influence which continues to turn insignificant. We thus continue to see the reappearances of additional determinants, the positive influences of *ROA*, *Rep Tax* and Div *Tax*, and negative influence of *Tax Interaction*. Thus, the corporate policy regarding large sized payouts is also influenced by the efficacy of the asset base's application for profit generation, and the tax framework. Overall, we conclude that when a firm is undertaking repurchases, their overall corporate policy is more determined by them than dividends, and the company's operating performance and the tax framework are key considerations in corporate decision-making.

## 5 Conclusion

We see that despite being dividend preferring, the UK has seen a consistent rise in repurchase undertaking over the past three decades. Our results indicate that corporate decision-making for dividends and repurchases differs primarily due to payout size, and secondarily due to payout type. This indicates that cash outflow to shareholders is the key consideration followed by the chosen route. Once the decision of distributing cash is decided, dividends and repurchases aggregately see heterogeneity in their determinants. Within dividends we see heterogeneity in the determinants of those that are small, medium and large sized; similar patterns are visible for repurchases as well. On comparison, only the determinants between small sized dividends and repurchases are homogeneous, thus making them substitutes, while medium and large sized are heterogeneous, thus making them independent. Our findings are relevant for future academics to use as a base, and extend them via using a new timeline post-2014 and/or replicate them for another country like the US. Practitioners can also capitalise on our findings to assess the decision-making of their portfolio constituents.

## Data availability statement

The authors do not have permission to share data.

## Additional information

No additional information is available for this paper.

## CRediT authorship contribution statement

**Adhiraj Sodhi:** Writing - review & editing, Writing - original draft, Validation, Methodology, Investigation, Formal analysis, Data curation, Conceptualization. **Aleksandar Stojanovic:** Writing - review & editing, Writing - original draft, Validation, Methodology, Investigation, Formal analysis, Conceptualization.

## Declaration of competing interest

The authors declare that they have no known competing financial interests or personal relationships that could have appeared to influence the work reported in this paper.

## Footnotes

1 We use this metric since dividends are annually determined by the company's net profitability.

2 A repurchase announcement states the % shares intended to be bought back via multiple transactions. Regulations allow for the repurchase price to vary by −/+5 % of the preceding five trading days. Thus, the value of the repurchase announced at the onset is an estimate. Further, distributing cash is the key motive for undertaking repurchases in the UK [32]. Hence, we tailor the dependent variable to capture these aspects.
